# Transcranial Direct Current Stimulation and Sports Performance

**DOI:** 10.3389/fnhum.2017.00243

**Published:** 2017-05-10

**Authors:** Dylan J. Edwards, Mar Cortes, Susan Wortman-Jutt, David Putrino, Marom Bikson, Gary Thickbroom, Alvaro Pascual-Leone

**Affiliations:** ^1^Non-Invasive Brain Stimulation and Human Motor Control Laboratory, Burke Medical Research Institute, Weill Cornell Graduate School of Medical Sciences, Cornell UniversityWhite Plains, NY, USA; ^2^Berenson-Allen Center for Noninvasive Brain Stimulation, Beth Israel Deaconess Medical Center, Harvard Medical SchoolBoston, MA, USA; ^3^School of Medical and Health Sciences, Edith Cowan UniversityPerth, WA, Australia; ^4^Department of Neurology, Weill Cornell Graduate School of Medical Sciences, Cornell UniversityNew York, NY, USA; ^5^Human Spinal Cord Injury Repair Laboratory, Burke Medical Research Institute, Weill Cornell Graduate School of Medical Sciences, Cornell UniversityWhite Plains, NY, USA; ^6^Department of Rehabilitation Medicine, Weill Cornell Graduate School of Medical Sciences, Cornell UniversityNew York, NY, USA; ^7^Burke Rehabilitation HospitalWhite Plains, NY, USA; ^8^Telemedicine and Virtual Rehabilitation Laboratory, Burke Medical Research InstituteWhite Plains, NY, USA; ^9^Department of Biomedical Engineering, City College of New York, City University of New YorkNew York, NY, USA; ^10^Institut de Neurorehabilitacio Guttman, Universitat Autonoma de BarcelonaBadalona, Spain

**Keywords:** tDCS, DIY, brain, sports, athletes

The application of transcranial direct current stimulation (tDCS) has moved from the laboratory to the wider community. This form of non-invasive brain stimulation has been shown in a number of controlled animal and human experiments, over nearly five decades, to modulate brain physiology, cognitive functions, and behavior. While its effects are variable across and within individuals, it is not unreasonable to state that tDCS harbors the potential to enhance executive and physical human performance. In a society increasingly driven to succeed with less effort, performance enhancement with an intervention that has an excellent safety record, is well tolerated, relatively inexpensive and readily available, is particularly appealing. Here, we offer a perspective on tDCS for the enhancement of physical performance in sport. The ethical and legal implications of the transition of tDCS from academic experimental work to general-public use, are discussed elsewhere (Janssens and Kraft, 2012; Bain et al., 2015; Fregni et al., 2015; Bikson et al., 2016a; Kuersten and Hamilton, 2016; Zettler, 2016).

We know from modeling (Datta et al., 2009; Luu et al., 2016), imaging (Baudewig et al., 2001; DosSantos et al., 2012; Jog et al., 2016), intra-cranial recording (Huang et al., 2017), and physiological studies (Nitsche and Paulus, 2000; Edwards et al., 2013; Strube et al., 2016) that the electrical current from tDCS can penetrate the skull to influence neural tissue and vasculature (Hamner et al., 2015). A good way to intuit that small amounts of current can transverse the skull is to recognize that the electroencephalogram (EEG) represents current passage in the reverse direction (Wagner et al., 2016). We also know that under laboratory conditions (Woods et al., 2016), the safety profile of tDCS is excellent, including in people with neurological and other disorders (Bikson et al., 2016b) although its safety for repeated and prolongued use in healthy individuals has yet to be confirmed (Wexler, 2016; Angius et al., 2017).

The spread of tDCS outside controlled laboratory conditions, fueled to some extent by media attention and high-profile users, has created concerns among some tDCS researchers. Recently we have seen the publication of an open letter recommending considerations for do-it-yourself (DIY) tDCS (Wurzman et al., 2016), including the involvement of healthcare professionals. A workshop hosted by the United States Institute of Medicine (IOM) addressed clinical and non-clinical applications of tDCS, including available evidence, safety, and ethics (Bain et al., 2015).

While tDCS can broadly modulate brain activity, and is considered safe within accepted boundaries, it remains to be conclusively determined whether it can improve sports performance at an elite level. The ability to optimize muscle control and maximize speed, power or duration is crucial to many sports, as is training and motivation (Crewther et al., 2016). In pursuit of excellence, athletes already use holistic approaches that directly or indirectly influence the brain. Some of these approaches include: meditation and visualization (Rich et al., 2016), and acupuncture (Ahmedov, 2010), which can have central effects (Zhu et al., 2015). Other holistic techniques include music to reduce the perception of physical effort (Jarraya et al., 2012) and psychological tools for motivation or harnessing placebo effects (Sabino-Carvalho et al., 2016).

Many athletes implement at least one of these tools; which, while not scientifically proven in all cases, are considered safe. tDCS may be yet another example (Figure 1).

**Figure 1:**
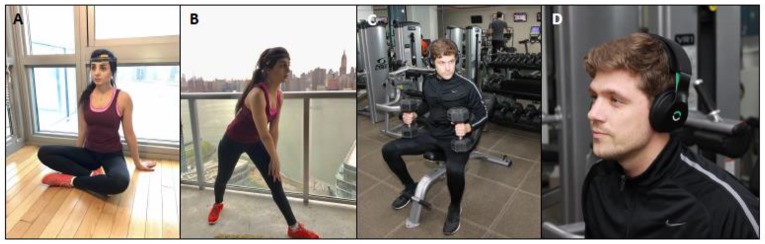
**Examples of tDCS device being used in sports training. (A,B)** Caputron tDCS device. **(C,D)** Halo tDCS device. Devices are typically used for 20 min before intensive training when motion is minimized, then removed when intensive physical training begins, comparable to timing in clinical rehabilitation.

Okano et al. studied the effects of 20 min of tDCS with the anode over the left temporal cortex (T3) on trained cyclists (Okano et al., 2013) during an incremental cycling test, and found significantly improved peak power, as well as reduced heart rate and perception of effort at submaximal workloads. Clarke et al. evaluated the effects of tDCS on a perceptual-learning paradigm (object detection in a simulated combat environment), showing significant enhancement of threat-detection accuracy with tDCS with the anode over the right inferior frontal cortex (Clark et al., 2012). In both cases, performance benefits were at least partially attributed to the effects of tDCS on perception (reduced fatigue and improved threat detection). Angius et al. (2016) likewise reported reduced perception of effort and increased endurance in 9 cyclists following anodal stimulation of the motor cortex (M1) when the cathode was placed on the contralateral shoulder but not when placed over the prefrontal region. Similarly, Borducchi et al. (2016), found that elite athletes gained a potential competitive advantage in cognitive performance and mood elevation, with 2 mA of tDCS with the anode over the left dorsolateral prefrontal cortex. By contrast, Flood et al. (2017) noted that while high definition tDCS targeting the sensorimotor cortex reduced perception of pain during fatiguing lower limb exercise in 12 subjects, there was no significant effect on muscle endurance or maximal production of force (Flood et al., 2017). There have been further positive (Cogiamanian et al., 2007; Abdelmoula et al., 2016) and negative (Kan et al., 2013; Muthalib et al., 2013) reports on the effects of tDCS with the anode over M1 on isolated muscle groups, such as the elbow flexor, and it is important to consider how post-exercise measurement of a unique muscle group in non-athletes might relate to conditions of athletic performance (Angius et al., 2017). Uncontrolled application of tDCS includes the US Olympic ski team (Reardon, 2016), top-tiered NBA team (Mansfield, 2016) and gamers (Falcone and Parasuraman, 2012; Jarrett, 2016). No doubt, the performance of these pioneering athletes will be followed carefully.

In a recent paper on athletic performance *stagnation*, Berthelot et al. (2015) questioned the extent to which athletic capabilities would progress beyond the boundaries of human physiological limitations. They conjectured, however, that technological breakthroughs might mitigate factors that limit physical performance. One such potential factor is perception of effort, which can be modulated by tDCS over M1, resulting in reduced perception of effort and greater endurance (Vitor-Costa et al., 2015). Fatigue contributes not only to reduced muscular endurance (Williams et al., 2013), but can also impair decision-making, response time and skill (Rattray et al., 2015). In addition, tDCS can enhance motor learning (Reis et al., 2009) thereby increasing the benefit of practice and promoting better performance. It is possible to hypothesize the mechanisms of action of tDCS which could lead to athletic performance enhancement; however, such hypotheses need careful testing before broad adoption.

One challenge for research scientists is to determine the efficacy of tDCS in real-world sports performance, and assess its safety in the context of repeated use. Additional questions remain regarding results found in laboratory conditions vs. field events and in athletes vs. healthy non-athletes; or improvements in strength vs. endurance, and upper extremity augmentation with tDCS juxtaposed with tDCS enhancement to lower extremities. A challenge for sports authorities is to determine where tDCS supplementation fits into the regulatory framework at the elite level. In the meantime, it seems likely that tDCS will continue to be explored by competitive athletes looking for that elusive *edge*. In the presence of media attention (Dubljević et al., 2014; Batuman, 2015) and marketing, it is likely that tDCS direct-to-consumer usage will expand. This engenders engagement from the scientific community. When and how are we obligated to step in as scientists? Is it desirable to learn from uncontrolled adoption in a form of crowd-sourced science (see Wexler, 2016; Wexler and Hamilton, 2017)? To what extent are companies marketing consumer products for sports performance beholden to the scientific community? Notwithstanding these complex questions, ongoing controlled experiments of tDCS in sports performance is of high value.

## Author contributions

All authors listed, have made substantial, direct and intellectual contribution to the work, and approved it for publication.

## Funding

This work was supported by the NIMH, NINDA, and NICHD of the NIH, under award numbers R01HD069776, 1R01NS101362-01, 1R01MH111896-01, 1R01NS095123-01, 1R01MH109289-01. AP was partly supported by the Sidney R. Baer Jr. Foundation, the NIH (R01MH100186, R01HD069776, R01NS073601, R21 NS082870, R21 MH099196, R21 NS085491, R21 HD07616), the Football Players Health Study at Harvard University, and Harvard Catalyst|The Harvard Clinical and Translational Science Center (NCRR and the NCATS NIH, UL1 RR025758).

## Disclosure

The content is solely the responsibility of the authors and does not necessarily represent the official views ofHarvard Catalyst,Harvard University and its affiliated academic health care centers, the National Institutes of Health, or the Sidney R. Baer Jr. Foundation.

### Conflict of interest statement

AP serves on the scientific advisory boards for Nexstim, Neuronix, Starlab Neuroscience, Neuroelectrics, Axilum Robotics, Magstim Inc., and Neosync; and is listed as an inventor on several issued and pending patents on the real-time integration of transcranial magnetic stimulation with electroencephalography and magnetic resonance imaging. The other authors declare that the research was conducted in the absence of any commercial or financial relationships that could be construed as a potential conflict of interest.
